# Empathy is associated with older adults’ social behaviors and verbal emotional expressions throughout the day

**DOI:** 10.1038/s41598-024-82550-0

**Published:** 2025-01-02

**Authors:** Meng Huo, Kate A. Leger, Kira S. Birditt, Karen L. Fingerman

**Affiliations:** 1 Department of Human Ecology, University of California, Davis, One Shields Avenue, Davis, CA 95616 USA; 2 Department of Psychology, University of Kentucky, Lexington, KY 40506 USA; 3 Institute for Social Research, University of Michigan, Ann Arbor, MI 48109 USA; 4 Department of Human Development and Family Sciences, The University of Texas at Austin, Austin, TX 78712 USA

**Keywords:** Late life, Empathic, Linguistics, EAR, EMA, Ecology, Psychology

## Abstract

Empathy plays a crucial role in promoting older adults’ interpersonal experiences, but it remains unclear how these benefits of empathy occur. To address this gap, we examined associations between empathy and how older adults behave and express emotions during their daily interpersonal encounters. Participants included 268 adults aged 65+ (46% men, *n* = 124) from the *Daily Experiences and Well-being Study*. They reported background characteristics and empathy in baseline interviews and indicated interpersonal encounters every 3 hours across 5 to 6 days. Participants wore electronically activated recorders (EAR), an app that captured 30-second snippets of ambient sounds every 7 minutes. Verbatim transcripts were coded for positive and negative social behaviors (e.g., praise, complain) and text was analyzed via Linguistic Inquiry and Word Count (LIWC) software for verbal expressions of positive and negative emotions (e.g., happy, hope, hate, hurt). Multilevel models showed that greater empathy was associated with greater variety in positive social behaviors throughout the day. More empathic older adults expressed more positive emotions while engaging in positive behaviors and less negative emotions when engaging in negative behaviors. This study innovatively draws on naturalistic data to delineate how more empathic older adults may have more positive and less negative social experiences than their less empathic counterparts. Findings may inform interventions that can incorporate empathy training to target those at higher risk of poor interpersonal experiences and outcomes (e.g., social isolation).

## Introduction

Bonding with others is a fundamental human need that is vital to health and survival^1–3^. As the basis for social connection involves the ability to share and understand others’ emotions^4–6^, it is not surprising that empathy has received burgeoning attention in research^7,8^. Mounting evidence points out the crucial role that empathy plays in later life^9–12^, when older adults are motivated to pursue satisfying social experiences that can buffer effects of inevitable health declines^13–15^. Indeed, research suggests that empathy facilitates older adults’ social connectedness and mitigates distress when tensions take place^9,11,16^. It remains less clear, however, how these benefits of empathy occur as older adults navigate the social world. To address this gap in literature, the current study assessed naturalistic observations of older adults’ daily social lives and examined how empathy might affect the way older adults behave and express emotions during interpersonal encounters (i.e., social behaviors and verbal emotional expressions). We focused on personality-like trait empathy that varies across individuals so as to inform tailored interventions targeting older adults at higher risk for poor relationships and health outcomes.

Despite holding a consensus that empathy includes both emotional and cognitive components, scholars do not necessarily agree on the terms they use when examining this ability^17,18^. Here we defined empathy considering empathic concern and perspective taking—two components that received the most research attention and have been theoretically associated with altruistic motivations, prosocial behaviors, and positive relationship outcomes^19–21^. Empathic concern captures the extent to which older adults *feel* concerned about the welfare of others in need whereas perspective taking refers to “standing in others’ shoes” to *understand* their thoughts and feelings. These two components correspond to distinct neural activities^22^ but are theorized to act jointly. The empathy-altruism hypothesis regards empathic concern as an essential predictor of altruistic motivations, but the Russian Doll Model also highlights perspective taking as a more advanced cognitive process that guides socially appropriate behaviors^19–21^. Notably, empathy can also bring costs, such that people may internalize the distress that they share from others. Such internalized distress is a self-centered component of empathy that is typically associated with inappropriate social behaviors^23–25^. In this study, however, we focused on other-oriented components of empathy and considered a combination of empathic concern and perspective taking to assess empathy in older adults.

Empathy is central to successful social lives, which may be reflected in how individuals behave when interacting with others. Burgeoning empirical studies on older adults have shown that those who are more empathic engage in more prosocial behaviors (e.g., donate money, help others, deal with tensions cooperatively), display fewer aggressive acts (e.g., yell, fight), and maintain better-quality social ties^9,11,16,26–29^. More empathic older adults tend to report greater emotional rewards when interactions are pleasant or prosocial than do their less empathic counterparts and are typically buffered against distress during negative moments in their relationships^8,12,16,30^. Yet, most of these studies examined older adults’ financial donation to strangers in the laboratory or relied on their retrospective self-reports of behaviors and emotional experiences. These methods are reliable and valid in some contexts, but they are also subject to limitations and biases (e.g., retrospective bias, social desirability)^31^. Here, we extend the literature by deriving coding of older adults’ behaviors as they interact with others in naturalistic settings and examining the way empathy shapes these social behaviors.

The current study assessed both positive behaviors intended to improve relationships and negative behaviors that can harm relationships. We followed a prior study^32^ that coded adults’ daily moral behaviors using ecologically sampled data of ambient sounds (snippets of auditory data intermittently recorded)^32–36^. This method is non-obtrusive and allowed us to capture subtle behaviors that older adults were not aware of or did not recall in surveys. In the current study, positive social behaviors include showing affection, offering praise, and expressing gratitude, whereas negative social behaviors refer to making sarcastic comments or complaining. Drawing on theories and research on empathy, we expected that more empathic older adults would be more likely to engage in positive social behaviors and less likely to engage in negative social behaviors.

We also examined links between empathy and the variety of positive and negative social behaviors. Engaging in diverse behaviors (positive behaviors in particular) may indicate flexibility in coping and emotion regulation, which refers to the capacity to utilize various coping strategies in response to a changing environment^37–39^. We asked whether more empathic older adults engaged in a greater variety of positive behaviors than less empathic older adults when interacting with others, such that they tend to say thank you to a spouse while also showing strong affection. Such variety in positive social behaviors may facilitate the goal of high-quality social ties and enhancing others’ well-being.

Due to the scarcity of research, however, we did not specify a hypothesis regarding the link between empathy and the diversity of negative behaviors. More empathic older adults may tend to avoid negative social behaviors in general, but it is unclear whether such avoidance can be identified in the diversity of their behaviors.

Further, auditory data offer novel insights into verbal emotional expressions—how older adults verbalize their emotions when interacting with others^40^. Similar to social behaviors, emotional expressions have been associated with interpersonal outcomes (relationship satisfaction, relationship quality)^41,42^. Yet, most work has focused on the role empathy plays in recognizing others’ emotion expression (facial expressions, in particular)^43,44^. To the best of our knowledge, no study has yet explicitly linked older adults’ empathy to the emotions they express. Given the prosocial nature of empathy^19–21^, it is possible that more empathic older adults care more about others’ feelings and have greater motivations to behave pro-socially and reduce others’ distress in social contexts. That is, empathy may affect the emotion words that older adults use in social contexts. For example, older adults may express pride when offering praise and talk in irritation when complaining about offspring, but such co-occurrence of verbal emotional expressions and social behaviors likely vary by empathy and empathy-elicited prosocial motivations. Compared to their less empathic counterparts, more empathic older adults may use more positive emotion words (i.e., expressing more positive emotions) when showing affection but prefer to tone down their complaints when upset (i.e., expressing less negative emotions).

In sum, we present a multi-method study to objectively capture older adults’ social behaviors and emotional expressions throughout the day and examine the role empathy plays in those aspects of interpersonal experiences. We relied on behavioral coding, linguistic analysis, and ecological momentary assessments to test our hypotheses and explore the mechanisms through which empathy promotes older adults’ social lives.

The first set of hypotheses pertained to older adults’ empathy and social behaviors, considering the occurrence and variety of positive and negative behaviors. We expected that more empathic older adults would be more likely to engage in positive behaviors and show a greater variety of positive behaviors than less empathic older adults. In addition, we expected empathy to reduce the frequency, if not also the variety, of negative behaviors.

Our second set of hypotheses examined the moderating effect of empathy on the association between social behaviors and verbal emotional expressions throughout the day. We expected empathy to strengthen the association between positive behaviors and expressions of positive emotions but attenuate the association between negative behaviors and expressions of negative emotions. To reduce confounding effects in hypothesis testing, we adjusted for background characteristics associated with empathy and social experiences: age, gender, education, self-rated health, marital status, and minority status^8,27,45–47^.

## Results

Table 1 describes our sample of 268 older adults who provided a total of 4,634 ecological momentary assessments (EMAs) across 1,104 days. EMAs were completed every 3 h throughout the day; on average, each participant completed 17.29 EMAs (*range* = 1–25 EMAs) across 4.12 days (*range* = 1–6 days). We also obtained 117,145 sound files captured by the electronically activated recorders (EARs), which were transcribed for behavioral coding and text analysis. In this sample, a typical older adult was 74 years old, completed at least some college, reported good to very good health, and rated medium levels of empathy. In total, 46% of older adults were male, 59% were married, and 24% belonged to a racial/ethnic minority group.

**Table 1:** Sample descriptive characteristics (*N* = 268).

	M	SD
Age	74.06	6.43
Education	6.00	1.49
Self-rated health	3.63	1.00
Empathy	3.77	0.64
Diversity of positive social behaviors^a^	0.94	0.54
Diversity of negative social behaviors^a^	0.33	0.25
Positive emotional expressions^a^	1.73	1.17
Negative emotional expressions^a^	0.25	0.24
	*Proportion*	
Male	.46	
Married	.59	
Ethnic or racial minority	.24	

^a^Averaged across ecological momentary assessments per participant.

Older adults reported having any social encounter across 89% of EMAs (4,100 out of 4,634 EMAs). Corresponding to each of the 3-hour EMAs when social encounters were reported, we coded the EAR data to assess the occurrence and variety of positive social behaviors (e.g., showing agreement, showing affection, expressing gratitude, providing constructive support, offering praise) and negative social behaviors (e.g., offering destructive support, being sarcastic, complaining or criticizing). On average, each older adult engaged in positive behaviors in 52% of EMAs and had negative behaviors in 28% of EMAs. In terms of the variety of social behaviors, almost 30% of EMAs (*n* = 1,323) involved two or more positive behaviors and just 5% (*n* = 234) with two or more negative behaviors. We also ran text analysis to assess verbal emotional expressions. In general, older adults seemed to express high levels of positive emotions and low levels of negative emotions in each EMA.

We conducted preliminary analyses to examine the bivariate correlations between empathy, social behaviors, and verbal emotional expressions (see Table 2). We found that empathy was positively associated with the average diversity of positive social behaviors in each participant but not associated with negative social behaviors. Empathy was not directly associated with verbal emotional expressions.

**Table 2:** Bivariate correlations.

	1	2	3	4	5	6	7	8	9	10
1. Age	—									
2. Education	− 0.02	—								
3. Self-rated health	− 0.05	0.27***	—							
4. Empathy	− 0.03	0.09	− 0.01	—						
5. Diversity of positive social behaviors	− 0.20	0.07	0.17**	0.14*	—					
6. Diversity of negative social behaviors	− 0.19**	− 0.03	0.11	− 0.04	0.51***	—				
7. Positive emotional expressions	− 0.12*	0.08	0.10	0.07	0.57***	0.40***	—			
8. Negative emotional expressions	− 0.16**	− 0.13*	0.02	− 0.03	0.28***	0.52***	0.31***	—		
9. Gender	0.04	0.16**	0.05	− 0.16**	− 0.24***	− 0.16*	− 0.16**	− 0.03	—	
10. Marital status	− 0.20***	0.16**	0.03	− 0.09	0.06	0.02	0.04	− 0.04	0.42***	—
11. Ethnic or racial minority	− 0.13*	− 0.31***	− 0.30***	0.02	− 0.09	− 0.12	− 0.02	0.08	0.03	− 0.03

To test our hypotheses, we estimated models using the 4,100 EMAs that involved at least one social encounter. In our first set of hypotheses, we expected greater empathy to be associated with more positive behaviors and fewer negative behaviors and considered both the occurrence and diversity of behaviors. Multilevel models revealed that more empathic older adults engaged in more diverse positive social behaviors than less empathic older adults during their interpersonal encounters throughout the day (*B* = 0.10, *p* = .046). Older adults’ empathy was not associated with the likelihood of engaging in positive (*Odds Ratio [OR]* = 1.19, *p* = .075) or negative social behaviors (*OR* = 0.93, *p* = .445). There was no association between empathy and diversity of negative social behaviors either (*B* = -0.02, *p* = .402). See Tables 3 and 4.

**Table 3:** Two-level logistic regressions predicting the likelihood of engaging in social behaviors from empathy. OR = odds ratios. Participants *N* = 268, EMAs *n* = 4,100 (we tested hypotheses selecting EMAs when social encounters were reported).

	Positive behaviors		Negative behaviors	
Variable	*OR*	*SE*	*OR*	*SE*
Fixed effects				
Intercept	—	—	—	—
Empathy	1.19	0.10	0.93	0.10
*Covariates*				
Age	0.98*	0.01	0.98**	0.01
Male	0.66**	0.14	0.71*	0.14
Education	1.03	0.05	0.95	0.04
Marital status	1.14	0.14	0.94	0.14
Health	1.16*	0.07	1.05	0.06
Minority status	0.93	0.16	0.65**	0.16
Random effects				
Intercept variance (level 2: participant)	0.66***	0.10	0.57***	0.09
-2 (pseudo) log likelihood	5,297.75		4,855.18	

*p < .05. **p < .01. ***p < .001.

**Table 4:** Two-level models predicting the diversity of social behaviors from empathy. Participants *N* = 268, EMAs *n* = 4,100 (we tested hypotheses selecting EMAs when social encounters were reported).

	Positive behaviors		Negative behaviors	
Variable	*B*	*SE*	*B*	*SE*
*Fixed effects*				
Intercept	1.38**	0.49	1.05***	0.24
Empathy	0.10*	0.05	-0.02	0.02
*Covariates*				
Age	-0.01*	0.01	-0.01**	0.00
Male	-0.29***	0.07	-0.08*	0.04
Education	0.00	0.02	-0.01	0.01
Marital status	0.05	0.07	-0.02	0.04
Health	0.08*	0.03	0.02	0.02
Minority status	-0.12	0.08	-0.09*	0.04
*Random effects*				
Intercept variance (level 2: day)	0.19***	0.02	0.04***	0.01
Intercept variance (level 2: participant)	0.00***	0.00	0.00***	0.00
Residual variance	1.09***	0.02	0.30***	0.01
-2 log likelihood	12,350.3		7,031.2	

*p < .05. **p < .01. ***p < .001.

**Fig. 1:**
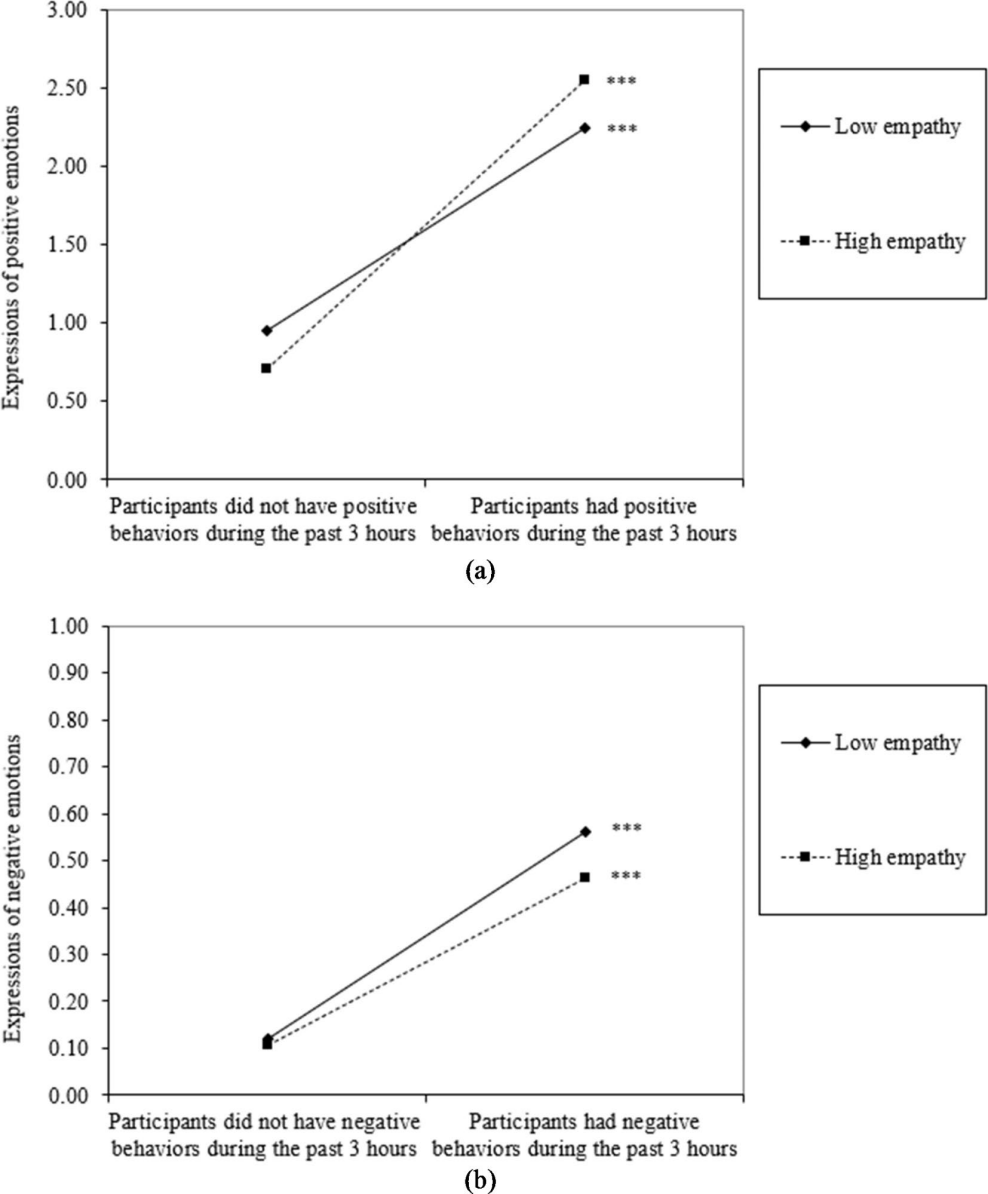
Interaction plots of empathy × (**a**) positive social behaviors and (**b**) negative social behaviors on verbal emotional expressions.

Our second hypothesis asked whether older adults’ empathy moderated associations between social behaviors and verbal emotional expressions. We included positive and negative social behaviors in the same models to adjust for potential co-occurrence of these behaviors. As expected, we observed two significant moderation effects. Participants expressed more positive emotions when engaging in positive social behaviors (*B* = 1.56, *p* < .001) compared to when they did not engage in these behaviors. Yet, this association varied by empathy (*B* = 0.43, *p* < .001), such that this association was stronger among more empathic older adults (*B* = 1.84, *p* < .001) vs. less empathic older adults (*B* = 1.29, *p* < .001). Likewise, we observed a link between negative social behaviors and expressions of negative emotions (*B* = 0.40, *p* < .001), also moderated by empathy (*B* = -0.07, *p* = .041). The association was weaker in more empathic older adults (*B* = 0.36, *p* < .001) than less empathic older adults (*B* = 0.44, *p* < .001). See Table 5; Fig. 1.

**Table 5:** Three-level models examining the moderating effect of empathy on the association between social behaviors and emotional expressions. Participants *N* = 268, EMAs *n* = 4,100 (we tested hypotheses selecting EMAs when social encounters were reported).

	Positive emotional expressions		Negative emotional expressions	
Variable	*B*	*SE*	*B*	*SE*
*Fixed effects*				
Intercept	0.83***	0.12	0.11***	0.03
Empathy	-0.19***	0.13	-0.01	0.03
Any positive social behavior	1.57	0.08	0.07***	0.02
Any negative social behavior	0.73***	0.08	0.40***	0.02
Empathy × Any positive social behavior	0.43***	0.12	0.03	0.03
Empathy × Any negative social behavior	0.01	0.12	-0.07*	0.03
*Covariates*				
Age	-0.00	0.01	-0.00	0.00
Male	-0.22	0.15	0.03	0.03
Education	0.05	0.05	-0.01	0.01
Marital status	0.01	0.15	-0.06	0.03
Health	0.07	0.07	-0.00	0.01
Minority status	0.04	0.17	0.03	0.03
*Random effects*				
Intercept variance (level 2: day)	0.73***	0.09	0.02***	0.00
Intercept variance (level 2: participant)	0.01***	0.00	0.00***	0.00
Residual variance	4.77***	0.11	0.34***	0.01
-2 log likelihood	18381.3		-297.7	

*p < .05. **p < .01. ***p < .001.

**Fig. 2:**
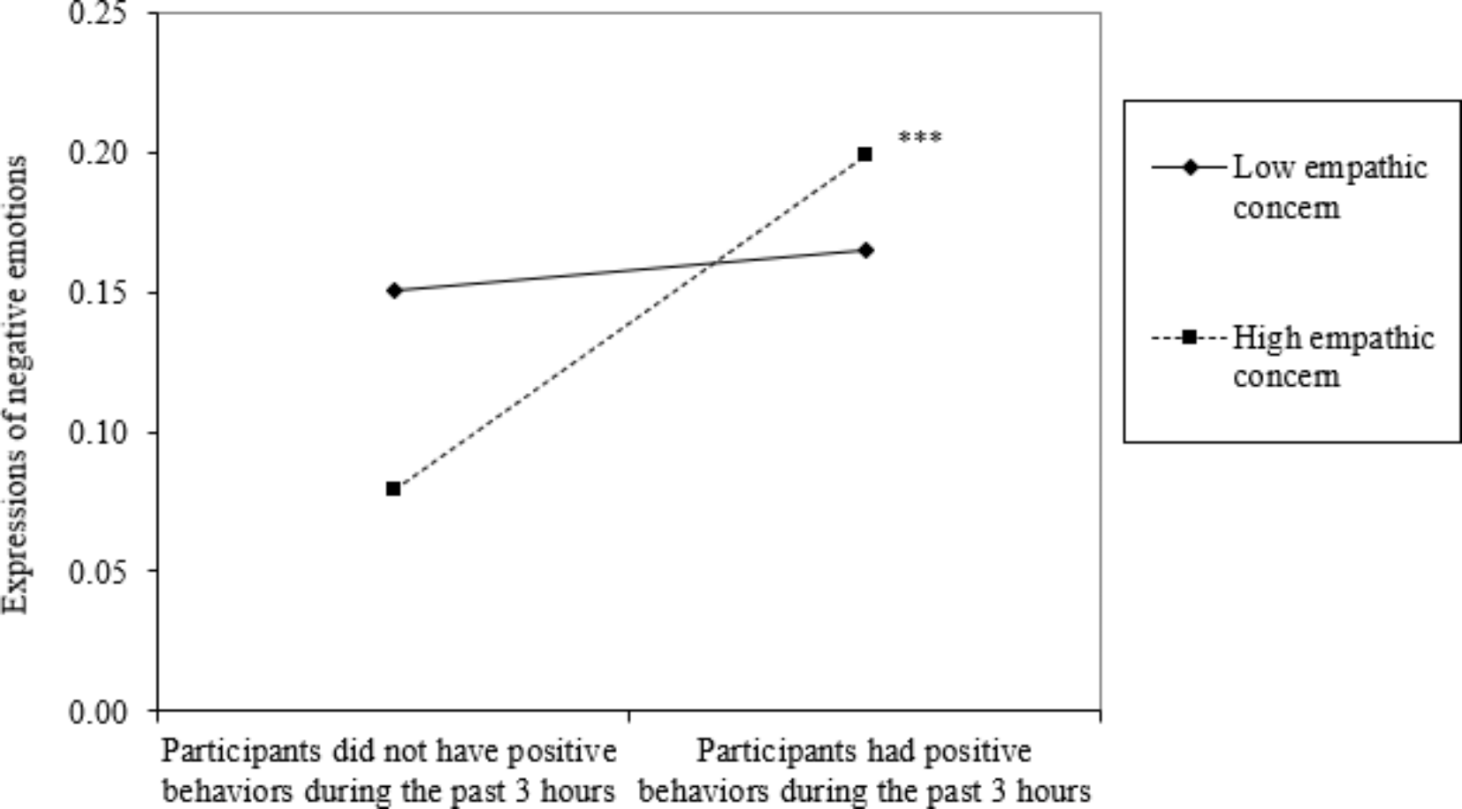
Interaction plot of empathic concern × positive social behaviors on verbal expression of negative emotions.

We then estimated post hoc tests examining empathic concern and perspective taking separately. Most results remained the same, with two exceptions. Although greater overall empathy was positively associated with the diversity of positive social behaviors, we observed no significant association involving either empathic concern or perspective taking. Additionally, we found that when empathic concern was high (*B* = 0.12, *p* < .001), positive behaviors were associated with more negative emotions expressed during interpersonal encounters; the association was not significant when empathic concern was low (see Fig. 2).

## Discussion

Empathy has long been considered vital in social contexts^5,7,19,20,48^, but the current study made unique contributions by revealing the role of empathy in older adults’ interpersonal experiences throughout the day. Utilizing behavioral coding and text analysis, we derived naturalistic observations of older adults’ social behaviors and emotional expressions. We found that more empathic older adults were not necessarily prone to behaving positively in social encounters, but they engaged in more diverse positive behaviors (e.g., show affection, express gratitude, offer constructive support) than less empathic older adults. Additionally, more empathic older adults expressed more positive emotions along with positive social behaviors and expressed less negative emotions when engaging in negative behaviors. This study provides empirical evidence in support of the theories of empathy and adds to prior work in the field. Findings offer novel insights into the mechanisms underlying the prosocial nature of empathy in late life, which may inform interventions intended to promote older adults’ social experiences and well-being.

It is somewhat surprising that empathy was not associated with a greater chance of behaving positively or a lower chance of behaving negatively. Yet, this finding is consistent with prior research that reported nonsignificant associations between empathy and the number of pleasant or stressful encounters in older adults’ daily lives^8,49^. The current study provides additional evidence that the way empathy promotes successful social lives may be more nuanced than simply exposing older adults to a greater number of positive experiences. Indeed, empathy seems to affect the diversity of older adults’ positive social behaviors throughout the day. A growing body of work has documented the psychological benefits of coping flexibility and emotion regulation variability—employing various strategies (e.g., rumination, avoidance, acceptance, problem solving, reappraisal) per specific social context^37,38,50^. The diversity of coded social behaviors may reflect variability in older adults’ behaviors addressing positive and negative moments in their relationships. As such, our findings suggest a potential role that empathy plays in promoting older adults’ adaptation to interpersonal challenges.

Further, we innovatively examined how empathy affected the association between older adults’ social behaviors and verbal emotional expressions. In short, engaging in social behaviors appear to co-occur with more positive and less negative emotional expressions in older adults scoring higher in empathy. The findings add to prior work that revealed how greater empathy in older adults was associated with more positive ratings of pleasant encounters and reduced stress during interpersonal tensions^8,16,49^. Indeed, a recent study revealed that even when couples were faced with a major stressor such as dementia, greater empathy was associated with more pleasant and less stressful feelings when people with early-stage dementia and spousal caregivers helped each other^30^. Based on observations of verbal expressions, it seems that empathy promotes quality rather than quantity of encounters, which in turn lead to better social experiences and well-being. Surprisingly, our post hoc tests seemed to indicate some costs of empathic concern, given that engaging in positive social behaviors was positively associated with expressing negative emotions among older adults with greater empathic concern. Yet, this finding should be interpreted with caution given the lack of more details in older adults’ social lives. For example, older adults tend to express negative emotions while discussing something stressful, but those with greater empathic concern are more likely to subsequently engage in positive social behaviors to address the tension. We call for more research that may draw on novel tools including sensors and observations to generate more informative observations of older adults’ interpersonal encounters, such as the sequence of social behaviors and emotional arousal as detected via voice analysis^51^.

There are limitations to this study that warrant consideration. We did not utilize all items from the widely used Interpersonal Reactivity Index^52^. Instead, we asked older adults to complete a shortened version to minimize their fatigue during a 2-hour interview, which is common when time or question quantity is constrained^53^. Moreover, the current study examined personality-like trait empathy with a focus on the two other-oriented components of empathy that received the most attention in research. Data on empathy that varies momentarily (e.g., empathic accuracy) or other components of empathy (e.g., personal distress) can be helpful for advancing our understanding of the role empathy plays in older adults’ daily social lives.

Another limitation involves reliance on transcripts of linguistic data, which do not capture the full encounter. Nonverbal behaviors such as hugs and facial expressions can also be prosocial and affect older adults’ interpersonal experiences^54^. For example, we did not find a significant link between empathy and the likelihood of engaging in any positive or negative social behaviors. It is possible that empathy affects nonverbal behaviors.

In addition, participants reported interpersonal encounters every 3 h throughout the day so the coded social behaviors and emotional expressions might not correspond to the same interpersonal encounters but rather could apply to all encounters that took place during the 3-hour interval. Video recordings of social interactions would provide additional information as we further examine the various aspects of interpersonal experiences, though obtaining such recordings throughout the day would introduce practical and ethical considerations.

Lastly, older adults in our sample did not engage in negative social behaviors often, which is consistent with theories and research on socioemotional aging suggesting that older adults have fewer negative social encounters and tend to rate their encounters as less negative^14,55^. The low variability in the diversity of negative behaviors could have contributed to its nonsignificant association with empathy. Future studies may consider more specific categorization to further differentiate among various negative social behaviors.

In sum, the current study presents ecologically valid measures of the central role empathy plays in older adults’ social lives. More empathic older adults displayed a greater variety of positive behaviors when interacting with others and they seemed to express emotions in those encounters in a more positive and prosocial manner. It is not yet clear whether these patterns of behaviors and emotional expression are essential in helping more empathic older adults navigate the social world more successfully and pleasantly. Yet, this study may set a foundation for future research to further explore the benefits of empathy in older age.

## Methods

### Sample and procedures

Data were from the *Daily Experiences and Well-being Study* (DEWS) conducted in 2016–2017. The study protocol and procedures were approved by the University of Texas at Austin Institutional Review Board (2015-02-0123) in accordance with the Department of Health and Human Services (HHS) regulations. All participants provided informed consent. The DEWS recruited 333 older adults aged 65 and over (65–92, *M*_*age*_ = 74.15) who resided in the community of the greater metropolitan Austin, Texas area (including urban, suburban and rural areas) and were not employed full time for pay^11^. We oversampled older adults in areas with high-density racial/ethnic minority population to obtain a diverse sample, with 33% of the sample identified themselves as ethnic or racial minorities (e.g., African Americans, Hispanic). We also recruited participants from a wide range of socioeconomic status; almost 50% of our participants did not have a college degree and our sample was only slightly more educated than the general population in Austin^56^.

Participants completed a 2-hour face-to-face interview (i.e., baseline interview) in their homes and were invited to participate in intensive data collection that lasted for 5 to 6 days (two weekend days and at least three weekdays). In the main interview, participants reported their background characteristics and rated their empathy. In the subsequent 5 to 6 days, participants were provided an Android mobile device to complete ecological momentary assessments (EMA) about their social experiences and well-being every 3 h throughout the day. An electronically activated recorder (EAR) was also installed on each Android device to collect 30-second snippets of ambient sound data every 7 min. Both EMA and EAR data were set up to be collected during waking hours as reported by participants. Participants received $50 for completing the main interview and an additional $100 for completing the daily intensive data collection.

Among the 333 participants who completed initial interviews, 283 were English speaking and provided EAR data that could be coded for everyday social behaviors. Of these 283 participants, 268 provided EMA data that could be matched with their EAR data based on timestamps. We extracted timestamps from EMA data corresponding to the 3-hour intervals when assessments were completed and then merged the timestamps to EAR data to identify all snippets that were recorded within each 3-hour interval. The final sample included 268 participants, who provided a total of 4,634 EMAs (*M* = 17.29, *range* = 1–25) across 1,104 days (*M* = 4.12, *range* = 1–6). Compared to the 65 participants excluded from the current study, the 268 participants were better-educated, healthier, and less likely to be a racial/ethnic minority.

## Measures

### Baseline interview: empathy

In the baseline interview, participants self-rated general empathy using items modified from the empathic concern and perspective taking subscales of the Interpersonal Reactivity Index (i.e., IRI)^11,26,49,52^. Each IRI subscale originally includes 7 items, but because empathy was measured as a part of a 2-hour interview, we instead included 4 items per subscale (i.e., 8 items in total) to avoid fatigue and reduce participant burden. Participants indicated the extent to which each item described them in general on a scale from 1 (*not at all*) to 5 (*a great deal*). During data collection, several participants reported confusion about reverse-scored statements such as “Sometimes I do not feel sorry for other people when they are having problems.” Thus, we excluded the reverse-scored items and averaged participants’ ratings across the remaining five items to measure overall empathy (*α* = 0.71). We also generated separate scores to assess empathic concern (*ρ* = 0.52; we calculated the Spearman-Brown coefficient for this two-item subscale^57^) and perspective taking (*α* = 0.78) for post hoc tests.

### EMA: interpersonal encounters

In the EMAs, participants reported on their interpersonal encounters every 3 h throughout the day. They separately indicated whether they had any encounter with (a) any of their 10 closest family and friends identified in the main interview using the social convoy measure^11,58^ and (b) anyone else. We combined those questions to assess whether participants had any interpersonal encounters during each 3-hour assessment (1 = *yes*, 0 = *no*). There were additional questions about those encounters (e.g., how pleasant each encounter was)^16^ and they were not included in the current study.

### EAR: positive and negative social behaviors

Social behaviors were coded using EAR data. A total of 117,145 EAR files containing sound were transcribed. For the purpose of this study, we trained a total of six undergraduate research assistants at the University of California, Davis to code verbalizations indicating social behaviors. Initially, four coders coded transcripts from five participants (#004 to #010; IDs are not consecutive) and resolved discrepancies through weekly discussions. To ensure that these coders had a clear and consistent understanding of the coding task, they coded transcripts from an additional 16 participants (#013 to #038). Two more coders joined the team and received training by coding the same transcripts from the first five participants (#004 to #010) and receiving feedback. All six coders coded transcripts from six more participants (#041 to #056). They were then divided into 3 pairs and coded another set of transcripts from 20 participants (#057 to #092). We calculated the inter-rater reliability for each of the social behaviors separately and present averaged reliability scores across pairs in Table 6. We took the sum for each of the behaviors per participant and estimated intraclass correlation coefficients given that the numbers of behaviors were continuous^59^. In total, data from 47 participants (18%) were coded by more than one coder and weekly discussions were held after the calculation of inter-rater reliability scores to generate the final set of codes of behaviors from these 47 participants. The coders independently coded transcripts from the remaining participants.

We began with the same list of behaviors as coded in a prior study examining daily moral behaviors in adults^32^ and then modified the list based on our data (see the coding scheme in Table 6). Positive behaviors included: (a) showing agreement, (b) showing affection, (c) expressing gratitude, (d) offering praise, and (e) offering constructive support. Negative behaviors included: (a) being sarcastic, (b) offering destructive behaviors, and (c) complaining/whining/criticizing/blaming. Behavioral coding was done for each transcript and was aggregated to reflect the occurrence of each behavior during the 3-hour intervals corresponding to EMA data. As for diversity of social behaviors, we calculated sums to measure the types of positive and negative behaviors that older adults engaged in during each 3-hour interval.

**Table 6:** Coding scheme for social behaviors and inter-rater reliabilty for each behavior. Interrater reliability was assessed as intraclass correlation coefficents given that behavior frequencies per participant were continuous variables. Reliability coefficients presented here were averaged across different pairs. For reliability coefficients lower than 0.80 (marked with asterisks), we re-estimated reliability coefficients based on coding that was done at a later stage, after coders had gone through rounds of discussions and when they were supposed to have better undersatnding of the coding scheme. Updated interrater reliaiblity coefficients 0.98 for offering praise, 1.00 for offering destructive support, and 0.98 for complaining, whining, criticizing, and blaming,

Behaviors (1 = yes, 0 = no)	Examples (adapted from EAR sound files)	Interrater reliability
*Positive behaviors*		
Show agreement	“Sounds good!” “That works for me” “good plan”	0.99
Show affection	“I love you.” “Girlie!”	0.96
Offer praise, make compliments	“That was delicious, Grace!” “Your outfit looks cute!” “She’s looking good today.”	0.78*
Show gratitude	“Thank you” “I appreciate you”	0.98
Offer constructive support (“Provide insight into the cause, offer a solution, solve the problem, or encourage further discussion”)	“Hey, please don’t leave that on the ground, someone may trip.”	0.96
*Negative behaviors*		
Offer destructive support (“Criticize or blame the support solicitor or offer inconsiderate help”)	“You need to do it this way or else it won’t work at all” “She ought to kick his ass”	0.84*
Be sarcastic, condescending, or arrogant	“Ha! You think that would work?” “Do I look like a person who would want to help you?”	0.94
Complain, whine, criticize, blame	“You just dropped that. Why did you do that?” “Honestly, Shane needs to get it together” “She’s so frustrating, she doesn’t know what she’s doing!”	0.87*

### EAR: verbal emotional expressions

Transcribed EAR data also were text-analyzed using the Linguistic Inquiry and Word Count (LIWC)^60^ based on established dictionaries of positive emotions (e.g., love, happy, good) and negative emotions (e.g., hate, worthless, hurt). We derived scores of positive and negative emotions to assess verbal emotional expressions in the transcripts. A score of zero reflects the absence of speaking during a certain snippet. These variables represent the level of verbal communication of emotions in each transcript that falls into predefined categories of positive and negative emotions^61,62^. We calculated mean scores of positive and negative emotional expressions for each 3-hour assessment time-wise matched with EMA data.

### Main interview: background covariates

Participants reported their age in years, gender (coded as 1 = *male* and 0 = *female*), education on a scale from 1 (*no formal education*), 2 (*elementary school*), 3 (*some high school*), 4 (*high school*), 5 (*some college/vocation or trade school*), 6 (*college graduate*), 7 (*post college but no additional degree*), to 8 (*advanced degree*), and physical health from 1 (*poor*), 2 (*fair*), 3 (*good*), 4 (*very good*), to 5 (*excellent*). Participants also indicated whether they were married, remarried, cohabitated, divorced, or single, which was dichotomized to reflect romantic relationship status (1 = *married/remarried/cohabitated* and 0 = *divorced/single*). We dummy-coded participants’ self-identified race (e.g., Native American, African American, Asian, White) and ethnicity (Hispanic or not) to measure racial/ethnic minority status (1 *= Hispanic*,* or non-White*, 0 = *non-Hispanic Whites*).

### Analytic strategy

We conducted preliminary analyses to test the bivariate correlations involving empathy, social behaviors, and verbal emotional expressions. Because social behaviors and emotional expressions were converted to reflect each 3-hour interval, we calculated mean scores for each participant before we ran the correlation tests.

Then we conducted hypothesis testing analyses as illustrated below.

We estimated multilevel models using SAS 9.4 to account for the nested structure of data, where the 3-hour assessment level (*level 1*) was nested within the day level (*level 2*), nested within the participant level (*level 3*).

Our first hypothesis involved the association between empathy and social behaviors throughout the day. Because the day-level random effects were zero (non-significant) for positive and negative social behaviors, we ended up dropping this level and estimating two-level models (3-hour assessments nested within each participant). Empathy was entered in the model as a *level 2* predictor to predict social behaviors as a *level 1* outcome. Positive and negative behaviors were examined as separate outcomes in models. When predicting the occurrence of positive and negative social behaviors (1 = *yes*, 0 = *no*), we used PROC GLIMMIX to run two-level logistic regressions and present odds ratios. Odds ratios indicate how the predictor empathy affects the likelihood of older adults engaging in positive and negative social behaviors, respectively. When predicting the diversity of social behaviors, the day-level random effects were significant. As such, we used PROC MIXED to run three-level models with continuous outcomes (3-hour assessments nested within days nested within participants).

We then tested our second hypothesis examining the moderating effect of empathy on the association between social behaviors and verbal emotional expressions. In this set of models, we kept the day level given significant random effects (i.e., we estimated three-level models). Positive and negative social behaviors were treated as *level 1* binary predictors (1 = *yes*, 0 = *no*) and emotional expressions were *level 1* continuous outcomes. We included cross-level interaction terms of social behaviors throughout the day (*level 1*) × empathy (*level 3*). Positive and negative behaviors were included in the same models to adjust for co-occurrence of these behaviors. Expressions of positive and negative emotions were outcomes in separate models.

Participant-level demographic characteristics were adjusted for in all models and continuous covariates were centered on the sample mean in the moderation tests. Significant interactions were explored in simple slopes analyses using ESTIMATE functions so as to better understand associations between predictors and outcomes at different levels of moderators (1SD above and below the mean level).

## Data Availability

The current study drew on data from the Daily Experiences and Well-being Study (PI: K. L. F.; https://www.icpsr.umich.edu/web/NACDA/studies/38570). The data we used for analysis and our analytic methods are described in detail in the text. Syntax will be made available to other researchers upon request.
